# Emphysematous Osteomyelitis Involving the Spine, a Case of a Rare Form of Osteomyelitis

**DOI:** 10.7759/cureus.41208

**Published:** 2023-06-30

**Authors:** Adil Khan, Farah N Zaidi, Maryem Anwar, Mashhood Ahmad, Anees Ur Rehman

**Affiliations:** 1 Internal Medicine, Khyber Medical College, Peshawar, PAK; 2 Medicine, Queen Elizabeth Hospital, King's Lynn, GBR; 3 Family Medicine, National Health Service (NHS), Slough, GBR; 4 Medicine, Ayub Medical College, Abbotabad, PAK

**Keywords:** vertebral body, medullary cavity of the bone, magnetic resonance imaging, staphylococcus aureus, emphysematous osteomyelitis

## Abstract

Gas-forming bacteria like *Staphylococcus aureus *(SA), along with the worrisome infections it causes, can lead to a fairly overlooked but lethal complication, called emphysematous osteomyelitis (EO). It is a condition characterized by the infection of bones complicated by the presence of gas-forming organisms. Here, we present a 50-year-old woman with insulin-dependent type 2 diabetes, who presented with cough, high-grade fevers, rigors, chills, and back pain from the last seven days. Laboratory tests revealed elevated inflammatory markers and a hyperosmolar hyperglycemic state. Sputum smear and blood cultures both reported the presence of SA. The presence of air foci in the third lumbar spine vertebra (L3) and the surrounding soft tissue was confirmed by computed tomography (CT). The posterior elements were spared, and the vertebral height and intervertebral disc were preserved. The extent of the damage done to the bone was analyzed by magnetic resonance imaging (MRI). The findings showed transverse relaxation time (T2) and short tau inversion recovery (STIR) sequences, consistent with the diagnosis of EO. She was put on strict glucose monitoring and a combination of intravenous antibiotics for three weeks followed by two weeks of oral therapy. Her symptoms improved with conservative management and reported no recurrence of any symptom of such type ever since.

## Introduction

With a mortality rate of 34%, emphysematous osteomyelitis (EO) can be regarded as an aggressive and a fatal condition [1]. In 1981, during an examination of computed tomography (CT) scans of some patients with osteomyelitis, Ram et al. found gaseous bubbles in the medullary cavity of the patients' bones. This led to the introduction of EO for the first time [2]. The gas found within the tissues was formed as a byproduct of the activities of anaerobic microorganisms [1]. EO, although rare, is a potential life-threatening condition, associated with the characteristic finding of gas locules in the bone in spite of having no direct communication with air outside [3].

The presence of gas, in vicinity and within the appendicular skeleton, virtually excludes all other conditions other than EO, but still, it may be safe to exclude certain differentials, such as trauma, post-surgical alterations, degenerative diseases, and neoplasms of the bone [4]. The organisms usually responsible for EO are *Fusobacterium necrophorum*, *Escherichia coli*, *Staphylococcus aureus *(SA), *Candida* spp., *Pseudomonas,* and *Klebsiella* [5].

## Case presentation

A 50-year-old woman presented to the emergency department with a seven-day history of fevers, rigors, chills, and lower back pain with no urinary or fecal incontinence. Her vitals were unstable, with a blood pressure of 85/65 mmHg, pulse of 120/min, body temperature of 38.8°C, respiratory rate of 28/min, and oxygen saturation of 92% (on room air). She was slightly drowsy, and her Glasgow Coma Score was 13. Her physical examination results revealed percussion pain in the vertebrae, dryness in the tongue and armpits, and tenderness in her back while sitting upright and on movement. Laboratory studies (Table 1) indicated leukocytosis (white blood cell count of 11,900/μL), an increased level of C-reactive protein (41.20 mg/dL), hyperglycemia (glucose of 900 mg/dL), and an increased level of glycated hemoglobin (13.5%), indicating uncontrolled diabetes mellitus. Other laboratory tests demonstrated only white blood cells and no ketones in the urine and serum, an effective plasma osmolality of 336 mOsm/kg, an arterial pH of 7.43, a serum bicarbonate level of 25.9 mEq/L, and an anion gap of 9 mEq/L.

**Table 1:** Laboratory values TLC: total leucocyte count, HB: hemoglobin, PLT: platelets, CRP: C-reactive protein, RBG: random blood glucose, HbA1c: glycated hemoglobin, HCO3: bicarbonates

Parameter	Day 1	Day 2	Day 3	Reference
TLC	11.9	12	11.3	4.5-11x10^9^/L
HB	12.3	12.6	13.0	Females: 12-15 g/dl
PLT	173	156	171	150-400x10^9^/L
CRP	41.20	42	50.1	Less than 5.0 mg/L
RBG	900 mg (50 mmol/L)	600 mg (33.3 mmol/L)	640 mg (35.5 mmol/L)	Less than 200mg/dL (11.0 mmol/L)
HbA1c	13.5%	13.4%	12.9%	Less than 6%
Plasma osmolality	336	367	309	275-295 mOsm/kg
HCO_3_	25.9	23.6	24	22-26 mEq/L
Ph	7.43	7.39	7.45	7.35-7.45
Anion gap	9	8.7	7.3	mEq/L
Blood urea	48.3	56.6	38.7	10-50 mg/dL
Creatinine	1	1.37	0.97	0.64-1.2 mg/dL
Sodium	146	123	140	135-150 mmol/L
Potassium	3.4	4	4.2	3.5-5.1 mmol/L
Chloride	107	111	118	96-112 mmol/L

**Figure 1:**
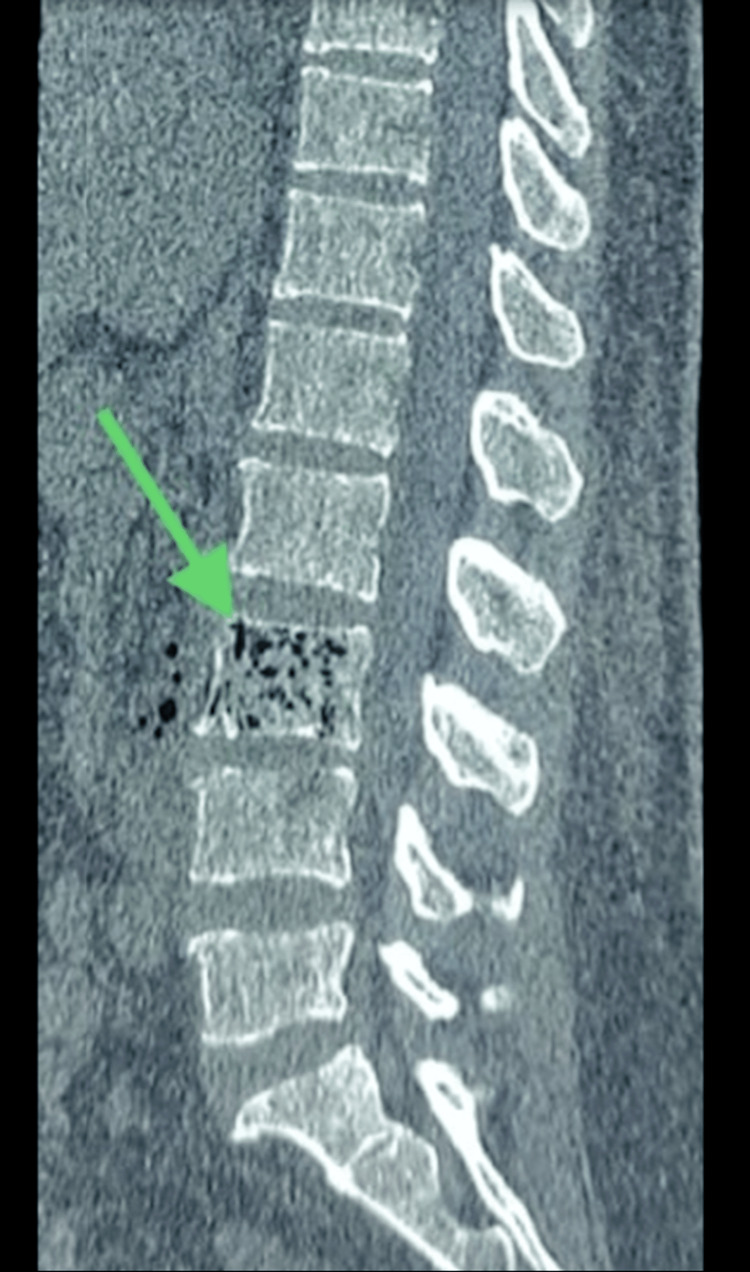
CT scan of the spine in the sagittal plane view shows intraosseous air foci at the L3, indicated by the arrow. CT: computed tomography, L3: third lumbar spine vertebra

**Figure 2:**
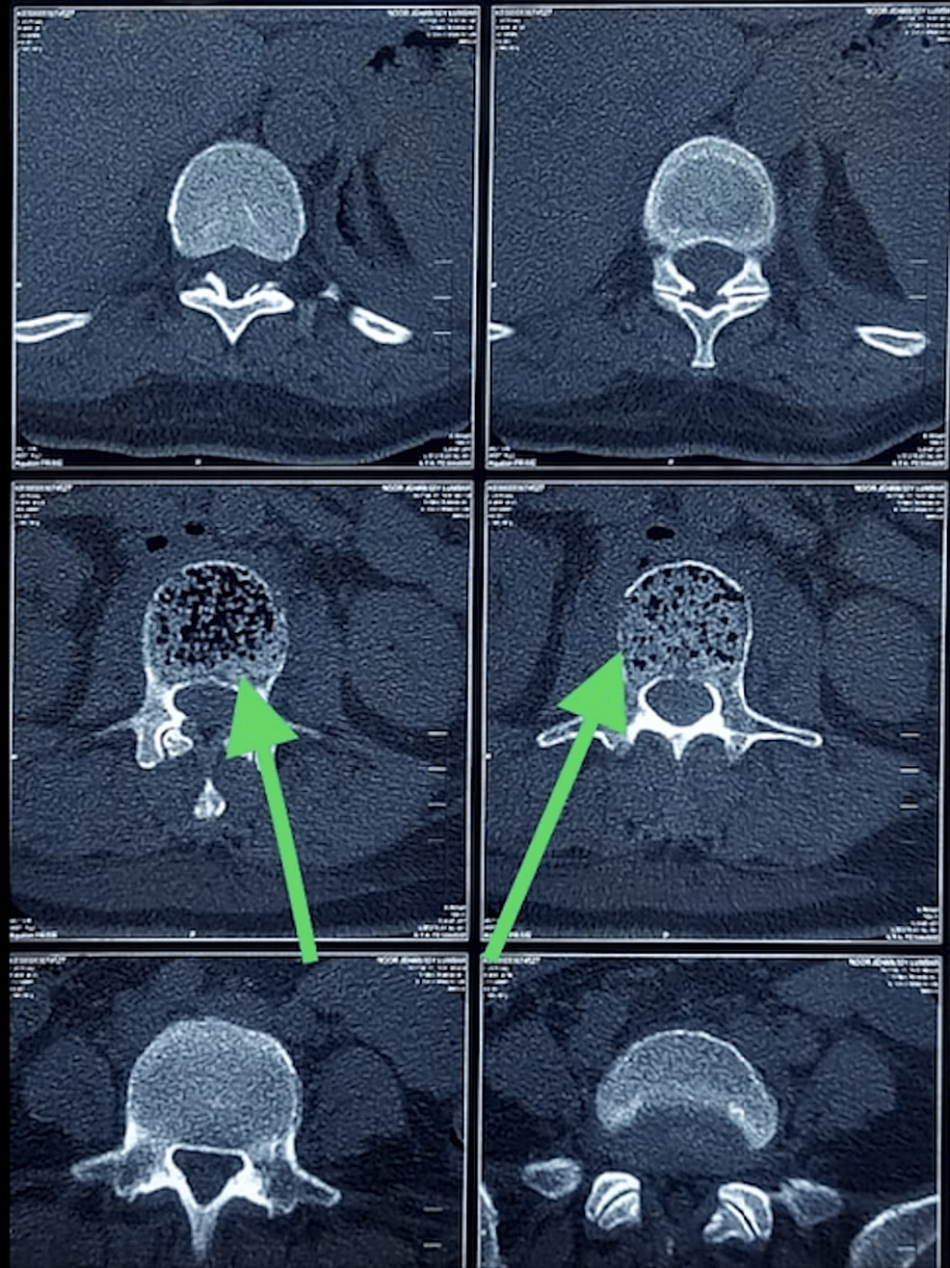
CT scan of the spine in the coronal plane view shows intraosseous air foci at the L3, indicated by the arrow. CT: computed tomography, L3: third lumbar spine vertebra

**Figure 3:**
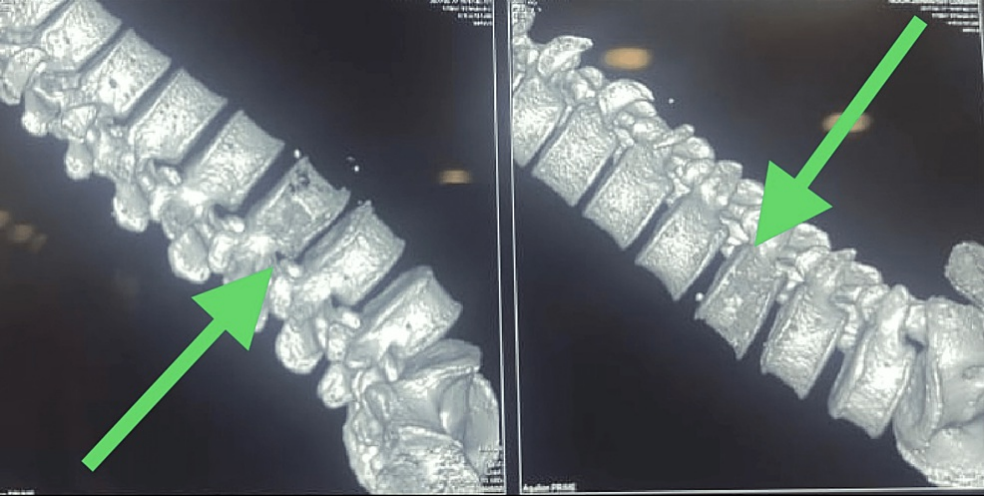
CT scan of the spine in the lateral plane view shows intraosseous air foci at the L3, indicated by the arrow. CT: computed tomography, L3: third lumbar spine vertebra

Initially, she presented with signs of sepsis, for which meropenem, being a broad-spectrum antibiotic, was started as an empiric therapy. A chest X-ray revealed infiltrates in both the lung fields, which were not consistent with an embolism. Blood cultures were performed, which were positive for SA. A transesophageal echocardiography was performed, which did not reveal any vegetations or regurgitations. These findings and the patient having no history of any intravenous drug abuse, implanted heart device, or poor dental health made bacterial endocarditis less likely. The patient, who had poorly treated diabetes and presented with SA bacteremia, SA pneumonia, and back pain, arose the suspicion of osteomyelitis. Workup for osteomyelitis included a negative X-ray of the spine but a positive CT scan (Figures 1, 2, 3) notable for emphysematous lesions of the third lumbar spine vertebra (L3) and surrounding erythema. Furthermore, it showed the posterior elements to be spared, and the vertebral height and intervertebral discs were preserved. 

**Figure 4:**
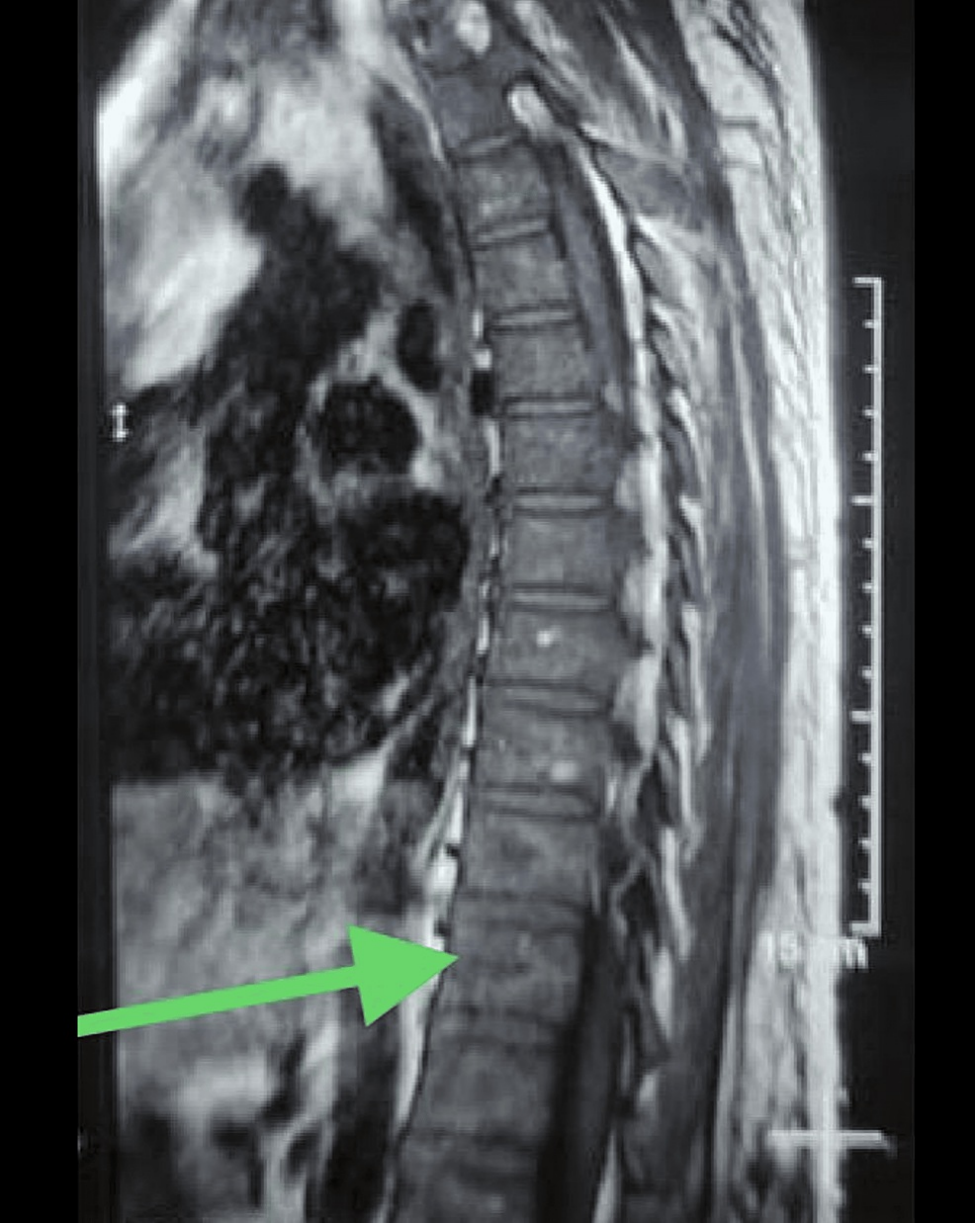
MRI of the spine showing the T2 and STIR sequences in the vertebral bodies and paravertebral soft tissue. MRI: magnetic resonance imaging, T2: transverse relaxation time, STIR: short tau inversion recovery

A magnetic resonance imaging (MRI) scan (Figure 4) was done to find the extent of the bone involvement, which confirmed the findings of the CT scan. It showed showed transverse relaxation time (T2) and short tau inversion recovery (STIR) sequences in vertebral bodies and paravertebral soft tissue, which was consistent with the diagnosis of EO.

The sustained SA bacteremia, which was evident from the repeat blood cultures, performed on different occasions, and the findings from the radiological imaging, led to the diagnosis of EO of the spine. The patient was admitted to the intensive care unit and treated for hyperosmolar hyperglycemic state and osteomyelitis with intravenous fluids and intravenous insulin. An antibiotic sensitivity test revealed the organism to be sensitive to oxacillin, trimethoprim-sulfamethoxazole, vancomycin combined with clindamycin, flucloxacillin, and linezolid. Although not the first line of antibiotics in this regard but in our setting, flucloxacillin and linezolid have been proven to be most effective in cases of severe infections with SA. Moreover, their cost effectiveness and easy availability make them the most preferred choice of drugs for this disease. Hence, an intravenous combination of flucloxacillin 1 gm and linezolid 600 mg was started twice daily. Neurosurgical and orthopedic consultations were obtained, and they advised conservative management. The patient recovered from the hyperglycemic state after three days. After 14 days, she was discharged home on the same intravenous antibiotics. She was kept on parenteral antibiotics for three weeks; then, on her first weekly follow-up, she was observed for her response toward the therapy. She was also evaluated for the development of thrombocytopenia and hepatic dysfunction, associated with the use of linezolid and flucloxacillin, respectively, but no problem was observed, and the patient was found stable enough to help herself to take the medicine orally and comply with the timing and dosage of the prescribed drugs. Hence, the intravenous antibiotic therapy was changed to oral flucloxacillin 500 mg, six hourly, and linezolid 600 mg, twice daily. The patient took these antibiotics for an additional two weeks. After an overall five weeks of antibiotic therapy, investigations were performed, which proved negative blood cultures for SA, chest X-ray with clear lung fields, and improved laboratory results, such as white blood cell count, improved C-reactive protein (CRP), and glucose levels (Table 2).

**Table 2:** Laboratory values TLC: total leucocyte count, HB: hemoglobin, PLT: platelets, CRP: C-reactive protein, RBG: random blood glucose

Parameter	Values	Reference
TLC	6.7	4.5-11x10^9^/L
HB	13.3	Females: 12-15 g/dl
PLT	280	150-400x10^9^/L
CRP	4.5	Less than 5.0 mg/L
RBG	199 mg (11.0 mmol/L)	Less than 200 mg/dL(11.0 mmol/L)

**Figure 5:**
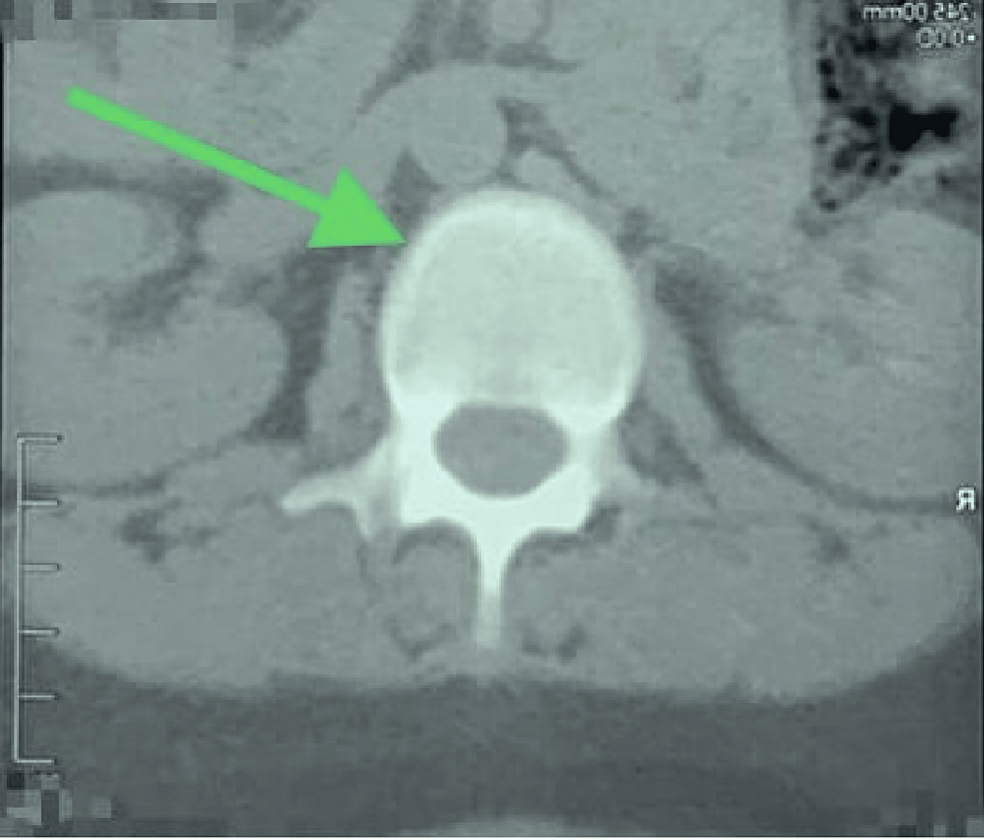
CT scan of the spine in the coronal plane view shows resolution of the intraosseous air foci at the L3, as indicated by the arrow. CT: computed tomography, L3: third lumbar spine vertebra

**Figure 6:**
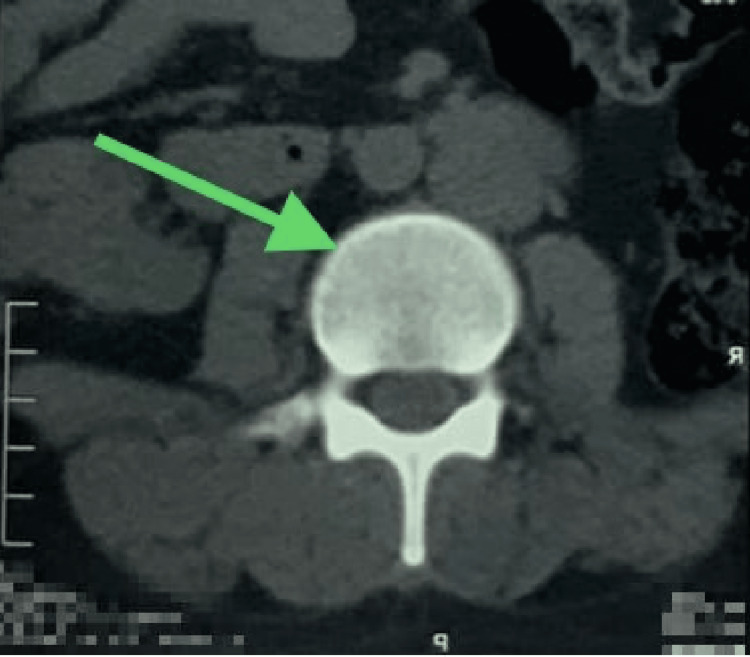
CT scan of the spine in the coronal plane view shows resolution of the intraosseous air foci at the L3, as indicated by the arrow. CT: computed tomography, L3: third lumbar spine vertebra

Moreover, a repeat CT scan was performed, which revealed resolution of gaseous locules (Figures 5, 6).

The patient remained asymptomatic for six months after completing the antibiotic treatment, which was evident on her monthly follow-ups. After six months, the follow-ups were discontinued. She did not report any recurrence of symptoms of such type ever since.

## Discussion

EO of the spine is a rare condition characterized by the presence of intraosseous gas in the vertebral column with the absence of direct bone-to-air connection [6]. This was first demonstrated in 1981, when Ram et al., while treating three patients with osteomyelitis, found locules of air in the femur on the CT scans of the patients. They could not establish any cause for it other than an infectious one [2].

As of now, only 48 cases of EO have been documented [7], and no specific sex or age tendency has been established. Our case involved a poorly controlled diabetic patient who presented with SA bacteremia, pneumonia, and EO. It is important to include SA bacteremia and bacterial endocarditis as a differential diagnosis of EO. In a review of previous cases of EO, only two cases were found to be due to SA [6]. The fact that this disease is uncommon may lead to late diagnosis. The high mortality rate (35%) of this illness can be attributed to the high virulence of gas-forming microorganisms, such as SA, and the patient's immune system, which may already be weakened by the presence of comorbid disorders (e.g., diabetes in our instance), frequently associated with patients with EO [8].

The review by Ryohei et al. summarizes the findings of the documented cases of EO. The condition occurs most commonly in patients who are immunocompromised: patients with diabetes mellitus (34.3%), malignancies (14.3%), or sickle cell anemia (8.6%) [9]. Our patient had severe uncontrolled diabetes, which led to the impairment of the patient’s immunity and predisposed her to a severe SA infection.

The timely diagnosis of EO is one of the most important steps in the management of this lethal complication. The role of imaging can be highly appreciated in this regard [10]. Hence, knowledge of the specific radiologic attributes of EO is of utmost importance. The most important is the “pumice stone appearance” due to the resemblance of air bubbles in the bone to the surface of the pumice stone [11]. Even before the development of symptoms, an MRI can detect degenerative changes of the bone, but, still, CT is regarded as the most sensitive for gas detection inside the bone and hence is the investigation of choice for EO [10].

The microorganisms first affect the surrounding tissues from which they make their way to the bone through the blood supply. However, some other routes of transmission have also been reported, such as an extension of the intra-abdominal infection, vertebral surgery, or infection involving soft tissues and the skin [12,13]. In our case, the infection was associated with pneumonia and its possible hematogenous spread to the vertebra. The presence of gas inside the bone is mostly due to the degenerative changes of the vertebrae or infections and less commonly due to osteonecrosis, trauma, or a malignant neoplasm [4]. Intraosseous gas in the intra-vertebral space is a common finding, associated with intervertebral disc degeneration, but the presence of gas inside the medullary cavity of the vertebra is pathognomonic for EO [14], which was the finding in our case. A wide variety of gas-forming anaerobes may act mutually, resulting in a polymicrobial infection, the most notorious being *Fusobacterium necrophorum*. However, any infection related to any single gas-forming bacteria can result in EO [5]. The common single agents include SA, *E. coli*, *Candida* spp., *Klebsiella,* and *Pseudomonas* species [5]. Our case had SA as the only organism that was isolated on a blood culture. As our patient had no history of any trauma or skin infection, the bone infection was due to the seeding of SA from a hematogenous source.

Treatments for EO, like any other form of osteomyelitis, require early diagnosis and prompt antibiotics. Some patients may require surgical debridement of necrotic bone [5], while some require surgical interventions for abscess development [9]. Hence, the treatment strategies include effective antibiotic therapies and surgical interventions as the main course of action. However, knowledge related to the guidelines and outcomes of the use of an antibiotic against its specific microbe is limited. As for surgical intervention, even though it has a good prognosis (which is evident from the cases it has been performed in), it still depends on whether it is required or not [9]. In our case, a surgical treatment was deemed unnecessary, and the patient was treated successfully with conservative management alone.

## Conclusions

EO is a rare entity that is often associated with diabetes. Knowledge regarding the association of diabetes and EO is well documented in the literature. In our case, antibiotic therapy alone was curative. However, we cannot say with confidence that this strategy would prove fruitful for any other cases of EO. Hence, it would be important to publish case reports of this entity in order to better establish treatment guidelines.

## References

[1] Sulyma V, Sribniak A, Bihun R, Sribniak Z (2020). Emphysematous osteomyelitis: review of the literature. Ortop Traumatol Rehabil.

[2] Ram PC, Martinez S, Korobkin M, Breiman RS, Gallis HR, Harrelson JM (1981). CT detection of intraosseous gas: a new sign of osteomyelitis. AJR Am J Roentgenol.

[3] Khanduri S, Singh M, Goyal A, Singh S (2018). Emphysematous osteomyelitis: report of two cases and review of literature. Indian J Radiol Imaging.

[4] Potocki J, Kaushik S, Mira JL (2003). Anaerobic osteomyelitis of femoral head with intraosseous, intra-articular, bursal and muscle pneumatosis. Skeletal Radiol.

[5] Lee J, Jeong CH, Lee MH, Jeong EG, Kim YJ, Kim SI, Kim YR (2017). Emphysematous osteomyelitis due to Escherichia coli. Infect Chemother.

[6] Luey C, Tooley D, Briggs S (2012). Emphysematous osteomyelitis: a case report and review of the literature. Int J Infect Dis.

[7] Sharma B, Lakhanpal V, Goyal A (2023). Extensive multifocal emphysematous osteomyelitis of spine: a rare case and a review of literature. Infect Disord Drug Targets.

[8] Mujer MT, Rai MP, Hassanein M, Mitra S (2018). Emphysematous osteomyelitis. BMJ Case Rep.

[9] Ono R, Uehara K, Kitagawa I (2018). Emphysematous osteomyelitis of the spine: a case report and literature review. Intern Med.

[10] Chamsi Basha A, Khalifa MA, Albadr F, Kaid J, Alsakkaf H (2021). Hip bone osteonecrosis with intraosseous pneumatosis after abdominal aortic aneurysm repair: a case of emphysematous osteomyelitis. BJR Case Rep.

[11] Small JE, Chea P, Shah N, Small KM (2022). Diagnostic features of emphysematous osteomyelitis. Curr Probl Diagn Radiol.

[12] McDonnell O, Khaleel Z (2014). Emphysematous osteomyelitis. JAMA Neurol.

[13] Masters EA, Ricciardi BF, Bentley KL, Moriarty TF, Schwarz EM, Muthukrishnan G (2022). Skeletal infections: microbial pathogenesis, immunity and clinical management. Nat Rev Microbiol.

[14] Aiyappan SK, Ranga U, Veeraiyan S (2014). Spontaneous emphysematous osteomyelitis of spine detected by computed tomography: report of two cases. J Craniovertebr Junction Spine.

